# Stress, burnout and resilience of teachers of students with emotional behavioural challenges

**DOI:** 10.1186/2193-1801-3-S1-O4

**Published:** 2014-12-04

**Authors:** Brenda Lai-kuen Lo

**Affiliations:** Principal’s Office, Youth College (Tseung Kwan O), Kragujevac, Hong Kong

## Background

The incidence of stress and burnout among teachers in Hong Kong has been increasing at an alarming rate in recent years [1, 2]. Studies on teacher stress and burnout have found that teachers of students with emotional behavioral challenges (EBC teachers) report more stress than general education teachers and are more likely to want to leave their position [3]. Studies conducted in Hong Kong [4] have also reflected the changing nature of the education system, in which the high expectations from stakeholders, inclusive education policy, increasing work load and the accreditation culture all put stresses on teachers. If left unresolved, teacher stress will have substantial negative effects on teachers’ physical and mental health, this will accelerate the turnover rate of teachers, and the whole educational system will be impaired and inefficiency will follow[5]. In view of the possible adverse consequences of teacher stress and burnout, certain measures must be undertaken to alleviate these problems.

This study examines the stress and burnout levels and their relation with the individual and organizational resilience of the teachers from seven Social Development Schools (special schools for maladjusted students who have varied EBC) in Hong Kong. The question of how these EBC teachers become resilient while teaching in the challenging school environment is explored, with a view to developing an explanatory model of the relation between stress, burnout and resilience and the factors and behaviors affecting resilience. Recommendations are drawn from this model on how teachers and school systems may act to alleviate the problem of burnout.

## Methods

A mixed-method approach was used that involved survey questionnaires and semi-structured interviews. All 146 EBC teachers of the seven Social Development Schools were invited to complete the questionnaire. Five existing instruments were adapted to form the questionnaire: the Source of Teacher Stress Scale, the Maslach Burnout Inventory-Educator Scale, the Teachers’ Sense of Efficacy Scale, the Resilience Scale, and the Collective Teacher Self-efficacy Scale. The analysis of the questionnaire data began with the development of descriptive statistics and moved to multiple regression (path) analysis to ascertain the relations among stress, burnout and resilience. In-depth interviews were conducted with 11 of the 106 respondents who rated themselves as resilient and who volunteered to participate in semi-structured interviews. Transcripts of the interviews were analyzed to identify and detail the significant factors and actions involved in the teachers’ resilience to burnout.

## Results

The results indicate that the stress level and burnout rate of the EBC teachers range from the moderate to high level. Lack of support, bring unprepared and overwhelmed by job responsibilities and the sense of dis-empowered are sources of stresses mentioned by the interviewees. There are correlations among stress, burnout and resilience. Rational coping behaviors and positive thinking strategies are personal resources that help teachers overcome stress and burnout. Individual resilience and organizational resilience both play significant roles in lightening the negative effects of stress and burnout. Strong support from the administrators has a supportive effect on the teachers.

## Conclusions

Recommendations are raised for teachers, school organizations and policy makers. For teachers, to enhance their own resilience and sense of self-efficacy is essential. For schools, work can be done to build a resilient school climate by designing local staff development programs to enhance their staffs’ personal growth and development; by offering training on positive thinking skills and rational coping strategies; by building a supportive mentoring system and positive school culture; and by allowing teacher participation in decision making by sharing teachers’ expectations about teaching and by facilitating the communication of teachers with organizational authority and among colleagues. For the policy makers, to ‘loosen and to slow down; to listen and to respond’ are suggested by the EBC teachers in this study.

The information obtained in this study will be used as reference to training needs for the development of a Life Skills Training Program for teachers. It is planned that the set of ‘Teacher Stress, Burnout and Resilience Questionnaires’ will be refined for future research, for both the special education sector and also for the vocational education and training sector to explore the needs of these teachers.
